# Supplementary material for: Carob Syrup: Prebiotic Potential of a Neglected Functional Beverage of Mediterranean Countries
Source: Foods. 2024 Dec 23;13(24):4172. doi: 10.3390/foods13244172 (PMC11675682; doi:10.3390/foods13244172)
Supplement: Supplementary file 1 [file foods-13-04172-s001.zip › foods-3357437-supplementary.pdf]

Supplementary material to:

Carob Syrup: prebiotic potential of a neglected functional beverage of Mediterranean countries

Lorenzo Nissen[1,2,*], Davide Addazii[1], Flavia Casciano[1], Francesca Danesi[1,2], Maria Teresa Rodriguez-Estrada[1,2], Dario Mercatante[1], Ben Ayache Siwar[3], Lotfi Achour[3], Anagnostis Argiriou[4] , Georgia Ayfandopoulou[5] and Andrea Gianotti[1,2].

[1]DiSTAL - Department of Agricultural and Food Sciences, *Alma Mater Studiorum* – University of Bologna, Viale Fanin 44, 40127 Bologna, Italy. lorenzo.nissen@unibo.it; davide.addazii@unibo.it; flavia.casciano2@unibo.it; francesca.danesi@unibo.it; maria.rodriguez@unibo.it; dario.mercatante2@unibo.it; andrea.gianotti@unibo.it

[2]CIRI - Interdepartmental Centre of Agri-Food Industrial Research, *Alma Mater Studiorum* – University of Bologna, Piazza G. Goidanich, 60, 47521 Cesena, Italy.

[3]BIOLIVAL - Bioresources: Biologie Integrative & Valorisation, Institut Supérieur de Biotechnologie de Monastir (ISBM), Av. Taher El Hadded BP74, 5000, Monastir, Tunisie. benayache.siwar@outlook.fr; lotfiachour@yahoo.fr

[4]UOA-FNS - Department of Food Science and Nutrition, University of the Aegean, University Hill, 81100 Mytilene, Greece. notis.argiriou@certh.gr

[5]CERTH/HIT - Centre for Research and Technology Hellas – Hellenic Institute of Transport, 6th km Charilaou – Thermi Rd., 57001, Thermi, Thessaloniki, Greece. gea@certh.gr

*Correspondence: lorenzo.nissen@unibo.it

Table S1. Primers pairs used in this study.

Strain target	Gene target	Sequence 5′-3′	Reference
Bifidobacteria mix	RecA r-DNA	fw: CGTYTCBCAGCCGGAYAAC<br>rev: CCARVGCRCCGGTCATC	[53]
Lactobacilli mix	16S V3/V4 r-DNA	fw: GCAGCAGTAGGGAATCT<br>rev: GCATTYCACCGCTACACA	[54]
Escherichia coli mix	16S V3/V4 r-DNA	fw: GTTAATACCTTTGCTCATTGA<br>rev: ACCAGGGTATCTAATCCTGTT	[55]

Table S2. Venn diagram of shared VOCs among all samples

**Compounds ID by NIST 11**
(-)-Epinephrine, tris(trimethylsilyl) ether
Benzaldehyde
Acetic acid
Benzoic acid
Butanoic acid
Butylated Hydroxytoluene
Eicosane
Ethanone, 1-(1H-pyrrol-2-yl)-
Furfural
Hexadecane
Octadecane
Phenol
Phenol, 2,4-bis(1,1-dimethylethyl)

Phenol, 2-chloro-4-(1,1-dimethylpropyl)-
Phenol, 4-(1,1,3,3-tetramethylbutyl)-
Propanoic acid, 2-methyl-
Tetradecane

Table S3. Occurrences of VOCs in different samples by Pairwise Intersection

**Compounds Identity by NIST 11**	**Present In**
(-)-Epinephrine, tris(trimethylsilyl) ether	CARS1, CARS2, CARS3, CARGR - CARCY, CARIT
1H-Pyrrole-2-carboxaldehyde	CARS1, CARS2, CARS3, CARGR - CARCY, CARIT
Acetic acid	CARS1, CARS2, CARS3, CARGR - CARCY, CARIT
Benzoic acid	CARS1, CARS2, CARS3, CARGR - CARCY, CARIT
Butanoic acid	CARS1, CARS2, CARS3, CARGR - CARCY, CARIT
Butylated Hydroxytoluene	CARS1, CARS2, CARS3, CARGR - CARCY, CARIT
Eicosane	CARS1, CARS2, CARS3, CARGR - CARCY, CARIT
Ethanone, 1-(1H-pyrrol-2-yl)-	CARS1, CARS2, CARS3, CARGR - CARCY, CARIT
Furfural	CARS1, CARS2, CARS3, CARGR - CARCY, CARIT
Hexadecane	CARS1, CARS2, CARS3, CARGR - CARCY, CARIT
Octadecane	CARS1, CARS2, CARS3, CARGR - CARCY, CARIT
Phenol	CARS1, CARS2, CARS3, CARGR - CARCY, CARIT
Phenol, 2,4-bis(1,1-dimethylethyl)	CARS1, CARS2, CARS3, CARGR - CARCY, CARIT
Phenol, 2-chloro-4-(1,1-dimethylpropyl)-	CARS1, CARS2, CARS3, CARGR - CARCY, CARIT
Phenol, 4-(1,1,3,3-tetramethylbutyl)-	CARS1, CARS2, CARS3, CARGR - CARCY, CARIT
Propanoic acid, 2-methyl-	CARS1, CARS2, CARS3, CARGR - CARCY, CARIT
Tetradecane	CARS1, CARS2, CARS3, CARGR - CARCY, CARIT
(-)-Norephedrine	CARS1, CARS2, CARS3, CARIT
1,2,5-Thiadiazolidine, 2,5-di-tert-butyl-, 1,1-dioxide	CARS1, CARS2, CARGR - CARCY, CARIT
1H-Indole, 1-methyl-2-phenyl-	CARS1, CARS2, CARS3, CARIT
2-Furanmethanol	CARS1, CARS2, CARS3, CARIT
2-Hexanol	CARS1, CARS2, CARS3, CARIT
2-Propanone, 1-hydroxy-	CARS1, CARS2, CARS3, CARIT
2H-Pyran-2-one, 5,6-dihydro-	CARS1, CARS2, CARS3, CARIT
3-Decen-5-one, 2-methyl-	CARS1, CARS2, CARS3, CARIT
4-Mercaptophenol	CARS1, CARS2, CARS3, CARIT
5-(2-Thienyl)pentanoic acid	CARS1, CARS2, CARS3, CARIT
Acetaldehyde	CARS2, CARS3, CARGR - CARCY, CARIT
Benzo[h]quinoline, 2,4-dimethyl-	CARS1, CARS3, CARGR - CARCY, CARIT
Butanoic acid, 2-methyl-	CARS1, CARS2, CARS3, CARIT
Carbon dioxide	CARS1, CARS2, CARS3, CARIT
Cyclohexasiloxane, dodecamethyl-	CARS1, CARS3, CARGR - CARCY, CARIT
Dodecanoic acid	CARS1, CARS2, CARS3, CARIT
Ethanone, 1-(2-furanyl)-	CARS1, CARS2, CARS3, CARIT
Heneicosane	CARS1, CARS2, CARS3, CARIT
Hexanoic acid	CARS1, CARS3, CARGR - CARCY, CARIT
Nonanoic acid	CARS1, CARS2, CARS3, CARIT
Octanoic acid	CARS1, CARS3, CARGR - CARCY, CARIT
Pentacosane	CARS1, CARS2, CARS3, CARIT
Phenol, 2,5-bis(1-methylpropyl)-	CARS1, CARS2, CARS3, CARIT
Tyramine, N-formyl-	CARS1, CARS2, CARS3, CARIT
1H-Indole, 5-methyl-2-phenyl-	CARS1, CARS2, CARIT
2(3H)-Furanone, 5-acetyldihydro-	CARS1, CARS3, CARIT
2(3H)-Furanone, 5-ethenyldihydro-5-methyl-	CARS1, CARS2, CARIT

2-Ethylacridine	CARS2, CARS3, CARGR - CARCY
2-Formylhistamine	CARS1, CARS3, CARIT
2-Phenylindolizine	CARS3, CARGR - CARCY, CARIT
2H-Pyran-2-one, 5,6-dihydro-6-pentyl-	CARS1, CARGR - CARCY, CARIT
3(2H)-Furanone, dihydro-2-methyl-	CARS1, CARS3, CARIT
3-Phenylindole	CARS1, CARS2, CARIT
5-Hydroxymethylfurfural	CARS1, CARS2, CARIT
Acetoin	CARS1, CARS2, CARIT
Benzenepropanal, 4-(1,1-dimethylethyl)-	CARS1, CARS2, CARGR - CARCY
Citric acid	CARS1, CARS2, CARS3
L-Alanine, methyl ester	CARS1, CARS2, CARS3
m-Anisic acid, cyclobutyl ester	CARS1, CARS2, CARS3
m-Cymene, 5-tert-butyl-	CARS1, CARS2, CARS3
meta-hydroxynorephedrine	CARS1, CARS2, CARS3
n-Hexadecanoic acid	CARS2, CARS3, CARIT
Orcinol	CARS1, CARS2, CARS3
Phenol, 4-fluoro-	CARS1, CARS3, CARIT
Propanamide, 2-hydroxy-	CARS1, CARGR - CARCY, CARIT
Propanoic acid	CARS1, CARS2, CARIT
Thiazole, 5-methyl-	CARS1, CARS3, CARIT
1-Hydroxy-2-butanone	CARS2, CARIT
1H-Isoindole-1,3(2H)-dione, 2-butyl-4,5,6,7-tetrahydro-	CARS1, CARIT
1H-Pyrrole, 2-ethyl-	CARS1, CARIT
2-Furancarboxaldehyde, 5-methyl-	CARS1, CARIT
4H-Pyran-4-one, 2,3-dihydro-3,5-dihydroxy-6-methyl-	CARS1, CARIT
5-Methyl-2-phenyl-2-hexenal	CARS1, CARS3
8-epi-.gama.-eudesmol	CARS1, CARS3
Bicyclo[2.2.2]oct-2-ene	CARS1, CARIT
Cyclotrisiloxane, hexamethyl-	CARGR - CARCY, CARIT
n-Decanoic acid	CARS1, CARS2
Propanoic acid, 2-hydroxyethyl ester	CARS1, CARIT
Tetrasiloxane, decamethyl-	CARGR - CARCY, CARIT

Table S4. List of VOCs identified by NIST 11 for one-way quantification heatmap

**Code**	RT (min)	#	**VOCs Identity by NIST 11**
A	19.118	1	n-Decanoic acid
B	20.465	2	Benzoic acid
C	20.756	3	Dodecanoic acid
D	25.359	4	n-Hexadecanoic acid
E	24.198	5	Citric acid
F	17.024	6	Octanoic acid
G	12.480	7	Butanoic acid
H	1.2967	8	Butanoic acid, 2-methyl-
K	14.895	9	Hexanoic acid
J	11.724	10	Propanoic acid, 2-methyl-
I	11.422	11	Propanoic acid
L	18.023	12	Nonanoic acid
M	10.260	13	Acetic acid

N	13.237	14	5-(2-Thienyl)pentanoic acid
O	21.842	15	Phenol, 4-(1,1,3,3-tetramethylbutyl)-
P	19.078	16	Phenol, 2,4-bis(1,1-dimethylethyl)
Q	19.292	17	4-Mercaptophenol
R	18.531	18	Phenol, 2-chloro-4-(1,1-dimethylpropyl)-
S	18.644	19	Phenol, 2,5-bis(1-methylpropyl)-
T	16.348	20	Orcinol
U	16.559	21	Phenol
V	14.755	22	Phenol, 4-fluoro-
W	12.826	23	2-Furanmethanol
X	20.967	24	5-Hydroxymethylfurfural
Y	16.786	25	1H-Pyrrole-2-carboxaldehyde
Z	10.401	26	Furfural
A1	15.862	27	Benzeneacetaldehyde, .alpha.-ethylidene-
B1	18.683	28	6-Chloro-2-methylquinoline
C1	17.303	29	5-Methyl-2-phenyl-2-hexenal
D1	1.995	30	Acetaldehyde
E1	11.843	31	2-Furancarboxaldehyde, 5-methyl-
F1	9.850	32	Benzenepropanal, 4-(1,1-dimethylethyl)-
G1	16.635	33	3-Decen-5-one, 2-methyl-
H1	8.143	34	Acetoin
K1	16.235	35	Ethanone, 1-(1H-pyrrol-2-yl)-
J1	2.632	36	Benzo[h]quinoline, 2,4-dimethyl-
I1	17.196	37	2(3H)-Furanone, 5-acetyldihydro-
L1	14.906	38	2H-Pyran-2-one, 5,6-dihydro-
M1	18.433	39	2(3H)-Furanone, 5-ethenyldihydro-5-methyl-
N1	18.779	40	2H-Pyran-2-one, 5,6-dihydro-6-pentyl-
O1	10.952	41	Ethanone, 1-(2-furanyl)-
P1	9.565	42	1-Hydroxy-2-butanone
Q1	7.451	43	3(2H)-Furanone, dihydro-2-methyl-
R1	19.022	44	4H-Pyran-4-one, 2,3-dihydro-3,5-dihydroxy-6-methyl-
S1	8.278	45	2-Propanone, 1-hydroxy-
T1	17.369	46	1H-Isoindole-1,3(2H)-dione, 2-butyl-4,5,6,7-tetrahydro-
U1	25.343	47	2-Formylhistamine
V1	25.580	48	Histidine, 1,N-dimethyl-4-nitro-
W1	24.370	49	Tyramine, N-formyl-
X1	4.302	50	(-)-Norephedrine
Y1	11.696	51	meta-hydroxynorephedrine
Z1	11.616	52	(-)-Epinephrine, tris(trimethylsilyl) ether
A2	6.895	53	Pyridine
B2	7.005	54	Picolinamide
C2	15.570	55	8-epi-.gama.-eudesmol
D2	11.233	56	m-Cymene, 5-tert-butyl-
E2	18.115	57	1H-Indole, 1-methyl-2-phenyl-
F2	9.909	58	Oxime-, methoxy-phenyl-
G2	22.622	59	2-Ethylacridine

H2	13.934	60	Carbon dioxide
K2	8.964	61	2-Hexanol
J2	10.833	62	Cyclohexasiloxane, dodecamethyl-
I2	17.261	63	Tetradecane
L2	13.356	64	Eicosane
M2	1.509	65	Dimethyl trisulfide
N2	6.047	66	Pentacosane
O2	7.197	67	2-Phenylindolizine
P2	8.364	68	3-Phenylindole
Q2	9.045	69	Propanamide, 2-hydroxy-
R2	9.218	70	Hexadecane
S2	9.671	71	Heneicosane
T2	10.763	72	Thiazole, 5-methyl-
U2	11.973	73	5-(2-Thienyl)pentanoic acid
V2	12.156	74	Octadecane
W2	13.156	75	1H-Inden-5-ol, 2,3-dihydro-
X2	13.237	76	Benzo[h]quinoline, 2,4-dimethyl-
Y2	14.420	77	Cyclotrisiloxane, hexamethyl-
Z2	14.658	78	Tetrasiloxane, decamethyl-
A3	15.090	79	Butylated Hydroxytoluene
B3	15.246	80	Lactose
C3	15.441	81	1H-Pyrrole, 2-ethyl-
D3	17.542	82	Bicyclo[2.2.2]oct-2-ene
E3	17.967	83	1,2,5-Thiadiazolidine, 2,5-di-tert-butyl-, 1,1-dioxide
F3	19.833	84	L-Alanine, methyl ester
G3	2.076	85	Sulfurous acid, dimethyl ester
H3	6.657	86	Propanoic acid, 2-hydroxyethyl ester
K3	9.331	87	m-Anisic acid, cyclobutyl ester
J3	25.089	88	1H-Indole, 5-methyl-2-phenyl-

Table S5. Significance of Spearman rank correlation.

NAME	Benzo	Dode	nHexade	Citric	Octa	Buta	Hexa	Nona	Orci	Phe4fl	Furanm	5HMF	Benzprop	Furan
PSL	0.384918	-0.60258	-0.46916	-0.42245	-0.28497	-0.00652	0.047539	-0.6826	-0.57736	-0.73058	-0.52613	-0.36733	0.415322	-0.85137
PSB	-0.37184	-0.14964	-0.27341	0.38202	0.017483	0.144379	-0.2126	-0.18616	0.43046	-0.09155	-0.03147	0.060447	0.332435	-0.14182
pH	0.932811	-0.10266	0.046073	-0.80564	0.524939	0.449837	0.508624	-0.10204	-0.88749	-0.08791	-0.10692	-0.07004	-0.42722	-0.12868
Aw	0.19589	0.227603	0.084418	-0.38552	0.555021	-0.07503	0.54903	0.304552	-0.16506	0.749128	0.282767	0.339071	-0.32901	0.73784
Lacto	0.481231	-0.41506	-0.27725	-0.46884	-0.3628	-0.0098	-0.02667	-0.53592	-0.65771	-0.68477	-0.38236	-0.28796	0.328209	-0.76798
Bif	-0.49651	0.128276	-0.07659	0.555863	-0.0779	0.165687	-0.17167	0.045936	0.593471	0.099818	0.246989	0.292619	0.325546	0.052378
Ecoli	0.197908	0.60524	0.637016	-0.03114	0.081732	0.192216	0.083193	0.63207	-0.07159	0.358783	0.493777	0.266896	-0.53456	0.428242
